# FEASIBILITY AND ACCEPTABILITY OF GAMIFIED CYCLING EXERCISE IN A LONG-TERM CARE HOME: A QUALITATIVE STUDY

**DOI:** 10.1093/geroni/igae098.3979

**Published:** 2024-12-31

**Authors:** Lillian Hung, Yong Zhao, Michelle Lam, Jamie lam, Arwen Fong, Tiffany Wu, Michelle Xiao, Haopu (Lily) Ren

**Affiliations:** University of British Columbia, Vancouver, British Columbia, Canada; University of British Columbia, Vancouver, British Columbia, Canada; University of British Columbia, Vancouver, British Columbia, Canada; University of British Columbia, Vancouver, British Columbia, Canada; University of British Columbia, Vancouver, British Columbia, Canada; University of British Columbia, Vancouver, British Columbia, Canada; University of British Columbia, Vancouver, British Columbia, Canada; University of British Columbia, Vancouver, British Columbia, Canada

## Abstract

Gamification can motivate older adults to exercise by transforming physical activities into enjoyable experiences. Incorporating gaming elements in cycling exercises can foster a sense of interest and achievement, potentially improving health outcomes. This study investigated the acceptability and feasibility of motivating residents living in long-term care homes with a gamified cycling exercise. Fourteen residents (13 living with dementia) completed a 4-week gamified cycling exercise twice a week. Safety during exercise was addressed by monitoring heart rate and symptom observation. With an interpretive description approach, we conducted interviews with residents and family members and focus groups with staff and leadership. The thematic analysis identifies three themes representing the feasibility and acceptability of gamified cycling exercise among LTC residents: ease of use and accessibility, physical and mental health benefits, fun engagement and community building. Future research should explore dementia-friendly design, culture-related game content, family orientation and engagement, group exercise and organization support. This study showed promising acceptability and feasibility of gamified cycling exercise in a long-term care home. Successful implementation relies on tailoring interventions to meet residents’ specific needs and preferences while acquiring rapport with interdisciplinary staff in the care home.

